# Determination of Osteocalcin Levels in Gingival Crevicular Fluid to Assess the Effectiveness of Laser Therapy in Patients with Orthodontic Treatment

**DOI:** 10.3390/biomedicines13112803

**Published:** 2025-11-17

**Authors:** Timea Dakó, Luminița Lazăr, Petra Șurlin, Dora-Maria Popescu, Anamaria Bud, Alexandru Vlasa, Mădălina Oprica, Sorina Mihaela Solomon, Ana-Petra Lazăr

**Affiliations:** 1 Department of Odontology and Oral Pathology, George Emil Palade University of Medicine, Pharmacy, Science, and Technology of Targu Mures, 38 Ghe. Marinescu Street, 540139 Targu Mures, Romania; timea.dako@umfst.ro; 2 Department of Periodontology, George Emil Palade University of Medicine, Pharmacy, Science, and Technology of Targu Mures, 38 Ghe. Marinescu Street, 540139 Targu Mures, Romania; alexandru.vlasa@umfst.ro; 3 Department of Periodontology, Faculty of Dental Medicine, University of Medicine and Pharmacy of Craiova, 2 Petru Rares Street, 200349 Craiova, Romania; popescudoramaria@yahoo.com; 4 Department of Pedodontics, George Emil Palade University of Medicine, Pharmacy, Science, and Technology of Targu Mures, 38 Ghe. Marinescu Street, 540139 Targu Mures, Romania; anamaria.bud@umfst.ro; 5 Immunology Laboratory, Center for Advanced Medical and Pharmaceutical Research, George Emil Palade University of Medicine, Pharmacy, Science, and Technology of Targu Mures, 38 Ghe. Marinescu Street, 540142 Targu Mures, Romania; madalina.nedelcu@umfst.ro; 6 Department of Periodontology, Grigore T. Popa University of Medicine and Pharmacy, 16th University St., 700115 Iasi, Romania; sorina.solomon@umfiasi.ro; 7 Department of Oral Rehabilitation and Occlusology, George Emil Palade University of Medicine, Pharmacy, Science, and Technology of Targu Mures, 38 Ghe. Marinescu Street, 540139 Targu Mures, Romania; ana.lazar@umfst.ro

**Keywords:** osteocalcin, bone remodeling, laser therapy, orthodontic treatment

## Abstract

**Background/Objectives**: Orthodontically induced bone remodeling is a complex process, driven by the interaction between osteoblasts, osteoclasts and various biochemical mediators, in response to mechanical forces applied to the teeth. Monitoring this process can be achieved by identifying biomarkers in gingival crevicular fluid (GCF), a dynamic and non-invasive method. Laser therapy, widely used in other medical fields for bio-stimulation and surgery, does not yet benefit from a standardized protocol in orthodontics. The aim of this study was to evaluate the advantages of using laser therapy during orthodontic treatment by analyzing osteocalcin (OC) in gingival crevicular fluid (GCF). **Methods**: Based on the inclusion and exclusion criteria, we selected 30 patients who presented dentoalveolar disharmony with crowding, who benefited from fixed orthodontic treatment, using edgewise brackets with the same slot size for all subjects. Laser therapy was performed randomly on one hemiarch (HL), right or left, for each patient, randomly chosen at time T0, after activation of the orthodontic appliance. On the other side, the control hemiarch (HC), the same protocol was followed, but without active light. Laser therapy was performed with a dental laser, with a power of 12 watts, setting the periodontology working mode. GCF was collected at baseline, before activation of the orthodontic appliance (time T0) and 14 days after its activation (time T1) from the control hemiarch (HC) and laser hemiarch (HL). Determination of OC levels, as a marker of bone apposition, was performed by the enzyme-linked immunosorbent assay (ELISA) method. To evaluate laser therapy, OC levels were assessed comparatively between HL and HC. **Results**: Comparing OC values at times T0 and T1 for HL, we obtained a statistically significant difference (*p* < 0.0001). No statistically significant difference was detected when comparing OC values in HC between T0 and T1 (*p* = 0.2422). A statistically significant difference was observed between HC and HL at T1 (*p* < 0.0001). **Conclusions**: The higher OC levels observed in the hemiarches where laser therapy was applied, compared to the controls, demonstrate its effectiveness as an adjuvant in bone remodeling during orthodontic treatment.

## 1. Introduction

Orthodontically induced bone remodeling is a complex biological process, regulated by the interaction between bone cells (osteoblasts and osteoclasts) and a series of local biochemical mediators, in response to the controlled application of mechanical forces on the teeth [1]. Under the action of these forces, bone resorption phenomena occur simultaneously on the pressure side and bone formation on the tension side of the periodontal ligament, thus contributing to the controlled movement of teeth during orthodontic treatment [2,3,4,5,6].

In the last decade, multiple research projects have focused on identifying biomarkers present in gingival crevicular fluid (GCF), with the aim of monitoring these processes dynamically and non-invasively. Gingival crevicular fluid is considered a fluid reflective of local tissue activity, and changes in its composition can provide relevant information about the state of the periodontium and bone remodeling processes [1].

Among the most commonly used biomarkers in this context is osteocalcin (OC), a marker of bone formation. Osteocalcin is a non-collagenous protein secreted by osteoblasts and integrated into the bone matrix, and its level in the gingival crevicular fluid is correlated with osteogenic activity [1].

In recent years, progress has been made in identifying adjuvant methods to accelerate tooth movement. Low-level laser therapy (LLLT) has been widely used in dentistry due to its multiple effects, such as inflammation reduction, modulation of cellular response, and biostimulator effects on tissue regeneration [7]. In orthodontics, LLLT is proposed as a non-invasive method to stimulate cellular activity in the periodontal ligament and alveolar bone. This effect is mediated by the increased expression of key factors such as RANKL, IL-1β, osteocalcin and TRAP, which regulate the activity of osteoclasts and osteoblasts [8,9,10]. The application of LLLT can lead to a significant increase in the levels of IL-1β and osteocalcin in gingival crevicular fluid during the initial phases of orthodontic treatment [8,9].

However, clinical data on the effectiveness of LLLT in orthodontics remain partly contradictory. Some studies reported significant acceleration of tooth movement and correlation with biochemical changes in GCF [11,12]. Other studies, however, have reported interindividual variation, lack of protocol standardization, and clinically insignificant results in some cases [10].

More recently, experimental studies have revealed the molecular mechanisms through which photobiomodulation influences osteogenesis, including activation of the BMP-2 pathway and the mitochondrial pathway [13]. Studies also demonstrated that laser therapy stimulates osteogenic differentiation of mesenchymal stem cells and increases Akt phosphorylation levels, thus supporting its potential in bone remodeling [14].

Although laser therapy is increasingly used in various medical branches due to its two major applications: biostimulation and surgery, in orthodontics, there is no protocol for its widespread use. Therefore, in this study, we aimed to evaluate the advantages and limitations of using laser therapy on bone remodeling processes during orthodontic treatment by analyzing osteocalcin levels in the gingival crevicular fluid.

Despite numerous studies investigating this subject, little is known about the real effects of LLLT on bone remodeling after orthodontic force application. The null hypothesis stated that the application of LLLT would produce no significant difference in osteocalcin levels between the laser-treated and control sites. Conversely, the alternative hypothesis assumed that LLLT would increase OC concentration, reflecting a stimulation of bone formation processes. By testing these hypotheses, the present research contributed to a broader line of investigation into biologically guided orthodontic treatments and the optimization of photobiomodulation parameters for enhanced tissue response. The study was designed to contribute to the ongoing efforts to establish evidence-based protocols for the use of photobiomodulation in orthodontics.

## 2. Materials and Methods

### 2.1. Study Design

The study had a split-mouth, randomized, double-blind design.

### 2.2. Patient Selection

From the adult patients with fixed orthodontic treatment, who came to CMI dr. Lazăr Luminița, between March 2023 and December 2023, 30 patients who met the following inclusion criteria were selected:

- Age between 20 and 50 years;
- Presence of dentoalveolar disharmony (DAD) with mild crowding (3–7 mm).

The exclusion criteria were as follows:

- Systemic conditions that have an impact on periodontal tissues (diabetes, immunological conditions, acute articular rheumatism, tuberculosis, etc.);
- Pregnancy or breastfeeding;
- Smoking;
- Treatment with antibiotics, vitamin D or Ca supplements in the last 3 months;
- Prior use of anti-inflammatory drugs (NSAIDs).

The patients were informed about the protocol, the fact that they could leave the study at any time and signed an informed consent.

### 2.3. Sample Size

For the primary comparison, we used a paired design (treated vs. control hemiarch). With α = 0.05, n = 30 pairs provide approximately 75% power to detect a medium effect size (Cohen’s d = 0.5).

### 2.4. Orthodontic Protocol

Patients presenting dentoalveolar disharmony with crowding were examined and received fixed orthodontic treatment using brackets with the same slot size for all subjects (0.22) (American Orthodontics, Sheboygan, WI, USA). The following sequence of arch wires was used: NiTi size 0.12 at the time of application of the orthodontic appliance and sizes 0.16 for the first activation, 0.16 × 0.16 for the second and 0.16 × 0.22 for the third (BioForce, Dentsply Sirona, Charlotte, NC, USA).

To simulate standard clinical conditions, light continuous forces ranging between 50 and 100 g were applied during each wire change. The force magnitude was measured and verified using a calibrated Correx tension gauge (Haag-Streit AG, Köniz, Switzerland) by recording the deflection of the archwire immediately after ligation. This ensured consistency of the applied forces across all subjects and prevented the use of extreme or outlier values.

### 2.5. Periodontal Protocol

Throughout the orthodontic treatment, we monitored the oral hygiene maintenance and periodontal status of the patients by recording the plaque index (PI), bleeding on probing index (BOP) and clinical attachment loss (CAL). Patients who presented inadequate hygiene at the activation sessions of the orthodontic appliance benefited from new training on oral hygiene measures. For patients who presented alterations in the periodontal status, we interrupted the active orthodontic treatment and instituted therapeutic measures corresponding to the degree of periodontal damage. The laser protocol and the immunoenzymatic analysis were performed only in conditions of a healthy periodontal status.

### 2.6. Randomization and Blinding

Hemiarch randomization was conducted using a computer-generated sequence, and allocation was concealed in sealed, opaque envelopes that were opened immediately before the application of the laser treatment. The laser therapy was administered by a member of the research team who was not involved in sample collection or data analysis.

To ensure proper blinding, both the patients and the investigators responsible for collecting and analyzing the gingival GCF were unaware of which hemiarch received the active laser treatment. All participants, as well as the operator, wore identical protective goggles that completely blocked visible light. The laser device emitted the same operational lights and sounds in both active and placebo modes, with the only difference being that no active laser emission occurred in the placebo mode.

As a result, patients, sample collectors, and data analysts remained blind to the treatment assignment, while the laser operator—who could not be blinded—was not involved in any assessment or analysis phases.

### 2.7. Laser Protocol

Another team member performed laser therapy on a hemiarch (HL), right or left, randomly selected at time T0, for each patient, after activation of the orthodontic appliance. Laser therapy was applied to a hemiarch at the level of the tooth selected for crevicular fluid collection and those located mesial and distal to it (HL). On the other side, on the control hemiarch (HC), the same protocol was followed, but without active light. During the irradiation, both the patient and the operator wore protective glasses. This protocol was applied at time T0, after the fixed orthodontic appliance activation session.

Laser therapy was performed with a dental laser (WISER3—Doctor Smile, Lambda S.p.A., Brendola, Italy), with a power of 12 watts, setting the periodontology working mode with the following specifications: 1 W, 450 nm, 1 cm^2^ beam area, and 15s exposure time per point.

Sites for laser application included the distobuccal gingival crest, the mesiobuccal gingival crest, a central spot on the buccal surface concerning each of the other sites, and two sites at the apex of the tooth parallel to points 1 and 2, respectively. The same spots were chosen for use on the palatal surface. Therefore, the laser was applied in contact with the tissue at 10 points per tooth. Each point was irradiated for 15 s (energy = 15 J; fluence = 15 J/cm^2^). Total energy per tooth per session was 150 J. The protocol included 1 session over 14 days, giving a cumulative dose per tooth of 150 J. CW emission corresponds to a 100% duty cycle. Although the device can deliver 12 W in surgical mode, in Periodontology mode, the parameters fall within LLLT standards.

### 2.8. Immunoenzymatic Analysis

GCF was collected from the tooth that will change its position, at baseline, before the activation of the orthodontic appliance (time T0) and 14 days after its activation (time T1) from the control hemiarch (HC) and laser hemiarch (HL), with special strips contained in the analysis kit. A strip for the analysis of OC in GCF was inserted buccally, in the tension area determined by orthodontic movement. Thus, for each patient, we used three strips, one at T0 and two at T1 (one for HC and one for HL).

Before collecting the GCF samples, the patients gently rinsed their oral cavity with water. Then the sampling area was gently dried with air, and the teeth were isolated using a cotton roll. Gingival fluid collection was performed using filter paper strips (Periopaper ProFlow Inc., Amityville, NY, USA), by inserting them into the gingival sulcus, at a depth of 1 mm, and maintaining them for 30 s or until the apical third of them was completely impregnated. Crevicular fluid volume was measured using a Periotron 8000 device (Pro-Flow Inc., Amityville, New York, NY, USA) that measures the change in capacitance on the filter paper.

The filter paper was inserted between the two plates of the device, and the device transmitted this change to a digital screen. These scores were converted into microliters using specialized software (mlconvert.exe—Ora Flow, Amityville, NY, USA). After each measurement, the Periotron electrodes were cleaned with a sterile compress soaked in saline to eliminate the risk of contamination. Strips contaminated with blood were excluded from the study.

After reading the strips, they were placed in an Eppendorf safe lock cryotube containing 250 microliters (μL) of phosphate buffer solution. These samples were stored in waxed boxes and kept at −80 °C until the completion of the harvests and their processing in the laboratory. The gingival crevicular fluid samples were not diluted before testing, according to the protocol.

**Figure 1:**
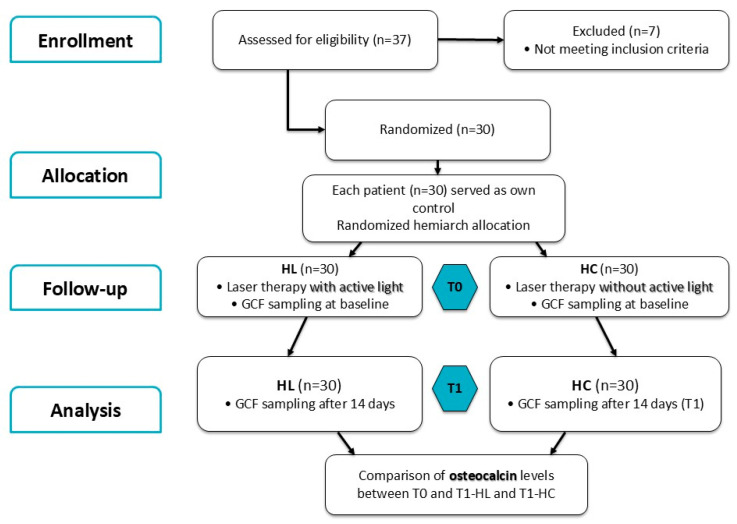
Flow diagram of the study following the CONSORT statement.

Determination of the levels of osteocalcin (ABclonal, Woburn, MA, USA), the marker of bone apposition, was performed by the enzyme-linked immunosorbent assay (ELISA) method, within the immunology laboratory of the CAMPhR of G. E. Palade UMPhsT of Targu Mures (Figure 1).

The reagents were brought to room temperature and prepared according to the working protocol. The gingival crevicular fluid samples were thawed, then vortexed (Vortex ZX4, Velp Scientifica, Usmate (MB), Italy) for 1 min and centrifuged (MiniSpin Centrifuge Eppendorf, Hamburg, Germany) for 2 min at 10,000 rpm.

According to the protocol, the wells of the plate underwent a washing step with a washing buffer.

In the first step, the standards and samples were added to the wells coated with a specific antibody for osteocalcin. During incubation, the OC present in the standard or sample bound to the immobilized antibody, forming an antibody–antigen complex. After the incubation period, the OC present in the standard or sample not bound to the fixed antibody was removed by washing with a washing buffer.

During the second step, a detection antibody specific for OC labeled with biotin was added, followed by an incubation period during which the detection antibody bound to the OC present in the standard and sample captured by the antibody fixed in the well, forming the antibody–antigen complex (the “sandwich” principle). After incubation, the unbound specific detection antibody was removed by washing.

In the third step of the method, the conjugate (streptavidin–HRP: streptavidin is a protein labeled with an enzyme—horseradish root peroxidase) was added, and during incubation, it bound to the specific detection antibody labeled with biotin. After the incubation period, the washing process was followed again, removing the unbound streptavidin.

Subsequently, the substrate (a solution containing a chromogenic compound—tetramethylbenzidine) was added, followed by an incubation period, which reacted with the conjugate, producing a color reaction (blue), depending on the amount of OC bound in the standard or sample.

After adding an acidic solution to stop the color reaction, the color turned from blue to yellow.

The absorbance of each well was read spectrophotometrically by the Dynex DSX automated ELISA analyzer (DYNEX Technologies, Chantilly, VA, USA); the analyzer software created a standard curve by plotting absorbance versus concentration for each standard. The OC concentration in the sample was determined by reading the absorbances for each well and interpolating onto the calibration curve, the protein concentration being directly proportional to the color intensity in the well. The concentrations obtained for OC were reported to the volume of phosphate-buffered saline (PBS) (dilution volume = volume of phosphate-buffered saline) and the volume of gingival crevicular fluid measured with the Periotron.

Performance characteristics:

- Kit sensitivity: The detection limit for osteocalcin is 0.1 ng/mL.
- Kit measurement range: Between 0.312–20 ng/mL. (There were no samples with concentrations outside the measurement range.)
- Kit precision: Intra-assay precision has a coefficient of variation below 10%; inter-assay precision has a coefficient of variation below 15%.

### 2.9. Laser Therapy Evaluation

To assess the effects of laser therapy, osteocalcin levels were assessed comparatively between HC and HL.

### 2.10. Statistical Analysis

All data were collected in Microsoft Excel spreadsheets (Microsoft Corporation, Redmond, WA, USA; 2018). Statistical analysis was performed in GraphPad Prism version 8.0.0 for Windows (GraphPad Software, San Diego, CA, USA). For each data group, descriptive statistics such as mean, standard deviation, median, minimum and maximum values were determined. Data normality was determined using the Kolmogorov–Smirnov test. Differences in osteocalcin values between baseline and T1 were evaluated using the Wilcoxon and paired t tests by comparing two groups. Friedman’s test was used to determine differences between the three groups by repeated measures. Pairwise post hoc analyses were performed using the Dunn test to determine which specific groups differed significantly from each other. The chosen significance level was set at 0.05.

The flow diagram of the study, following the CONSORT statement, is represented in Figure 1.

## 3. Results

A total of 30 patients (21 females and 9 males) aged between 20 and 50 years (mean age 24.6 ± 8.4) were included in the study.

The following formula was used to determine the OC values in the crevicular fluid:

$$Total\;protein\;amount\;\left(\mathrm{ng}/\mathrm{mL}\right)=\frac{Amount\;of\;identified\;protein\;(\mathrm{ng}/\mathrm{mL})\times Dilution\;volume\;(0.25\;mL)}{Crevicular\;fluid\;volume\;(mL)}$$

OC values are therefore expressed as concentrations (ng/mL) normalized to the GCF volume, allowing for inter-sample comparability regardless of the collected fluid quantity.

Because the OC data were not normally distributed, non-parametric tests were used for all comparisons. Therefore, results are reported as median and interquartile range (IQR). Mean ± SD values of osteocalcin (ng/mL) at baseline and T1 (for HC and HL) are provided in Table 1 for completeness and comparison with previous studies.

**Figure 2:**
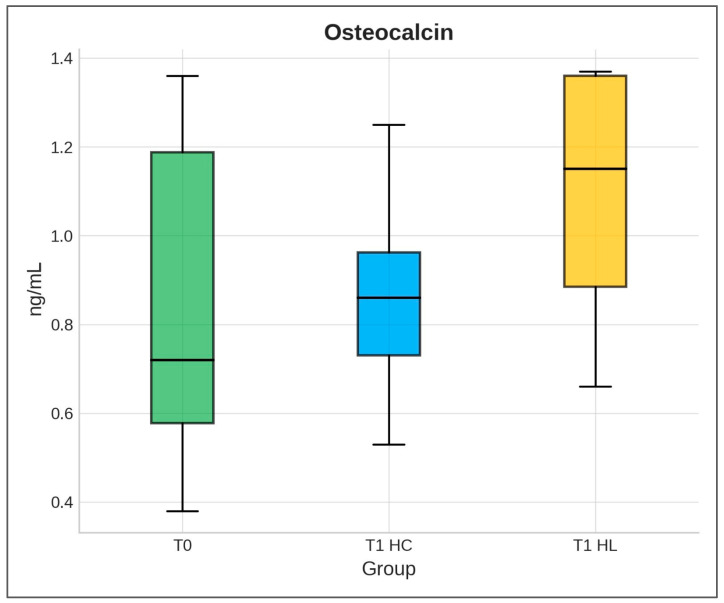
Distribution of osteocalcin level (ng/mL) at T0 and T1 for HC and HL. Values are expressed as median and interquartile range.

OC values at T0 and T1 for HL differed significantly (Wilcoxon test, *p* < 0.0001, median difference = 0.28, 95% CI: 0.21–0.34, n = 30). The effect size was large (r ≈ 0.88). No statistically significant difference was detected when comparing osteocalcin values in HC between baseline and T1 (Wilcoxon test, *p* = 0.2422, median difference = 0.0200, 95% CI: 0.10–0.50, n = 30). The effect size was small (r ≈ 0.18). A statistically significant difference was observed between HC and HL at T1 (Mann–Whitney U test, *p* < 0.0001, median difference = 0.2900, 95% CI: 0.230–0.350, n = 30). The effect size was large (r ≈ 0.89) (Figure 2).

**Figure 3:**
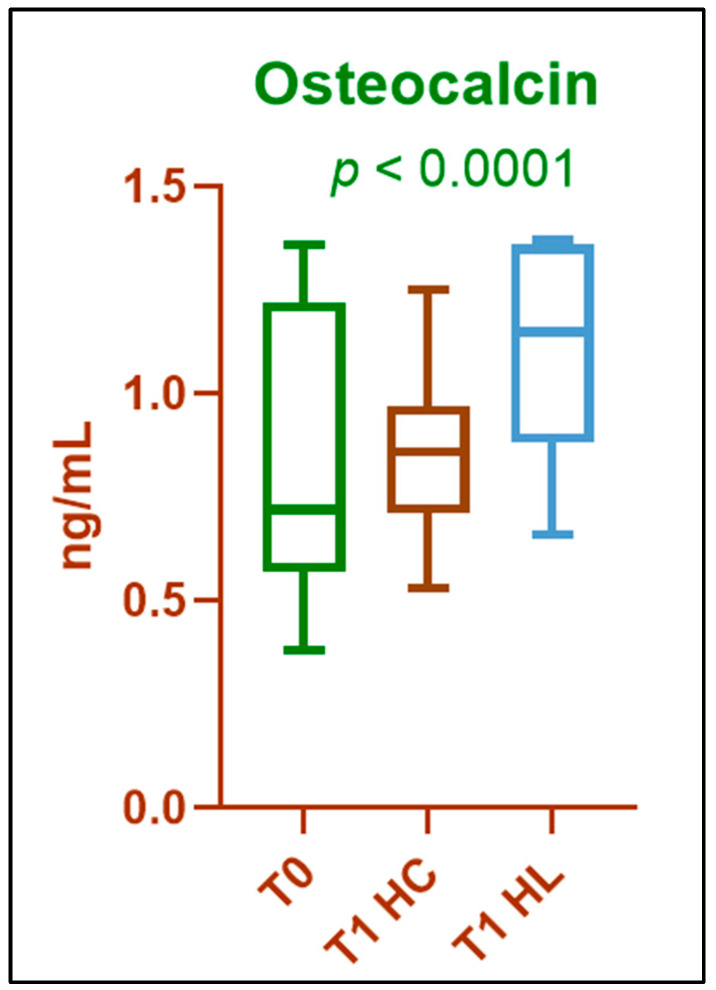
Comparison of osteocalcin values at baseline (T0) and T1 for HC and HL. Results are expressed as median and interquartile range; *p*-value was calculated using the Friedman test.

Friedman’s test showed significant differences between the three groups (*p* < 0.0001, Kendall’s W = 0.82, large effect size). Post hoc analysis (Dunn’s multiple comparisons) showed that osteocalcin levels were significantly higher in the lasered hemiarch compared to both baseline (*p* < 0.0001) and non-lasered hemiarch (*p* < 0.0001). The difference between baseline and non-lasered hemiarch was not significant (*p* = 0.59) (Figure 3).

## 4. Discussion

In the present study, in order to assess the effects of laser therapy during orthodontic treatment in adult patients, we comparatively evaluated the changes in osteocalcin (OC) levels in the gingival crevicular fluid in the laser-treated (HL) and control (HC) dental hemiarches.

Osteocalcin, synthesized by osteoblasts and released into the gingival crevicular fluid, is considered a marker of bone formation. This led us to collect samples for its evaluation from the tension zone of periodontal structures, secondary to orthodontic movements.

Previous studies have shown an increase in osteocalcin levels between days 7 and 14 after activation of the orthodontic appliance, with a peak around day 14, correlated with intense osteoblastic activity [1,15]. In a randomized split-mouth study, Yildirim et al. observed that osteocalcin levels in GCF, in the tension zone, are higher on day 14 (*p* < 0.05), during canine distalization with and without piezocision acceleration [16].

Based on these observations, we chose to collect samples 14 days after activation of the orthodontic appliance, at which time the levels of this marker would be higher in GCF.

Consistent with previous studies, our findings indicate that 14 days after the start of orthodontic treatment (T1), osteocalcin levels increased in most patients compared to T0, in both control (HC) and laser-treated (HL) hemiarches. This reflects elevated biochemical activity within the bone and an increased rate of bone turnover.

However, there are also studies according to which the level of osteocalcin in GCF does not change during orthodontic treatment. In a study by Alnazeh et al., osteocalcin levels in GCF were not significantly different at baseline and after 4 weeks (*p* > 0.05) in both fixed orthodontic and Invisalign patients [17].

Determination of bone markers in gingival crevicular fluid may be an objective method for monitoring the progress of orthodontic treatment and the effects of adjuvant therapies, such as laser therapy. However, it is important to emphasize that the levels of these biomarkers may also be influenced by factors such as gingival inflammation, poor oral hygiene, type or stage of orthodontic treatment.

In our study, the minimum value recorded for OC was 0.38 (ng/mL) at time T0, and the maximum was 1.37 (ng/mL) at time T1, in HL.

Some studies have reported that OC levels in GCF in periodontally affected areas are higher than in healthy areas [18,19]. When small orthodontic forces are applied, inflammatory mediators are released, similar to what occurs during periodontal diseases. Several studies support the fact that orthodontic forces trigger the release of inflammatory mediators and signaling molecules involved in periodontal tissue remodeling. Thus, IL-1β, TNF-α, and PGE2 increase significantly in the gingival crevicular fluid in the first 24 h after orthodontic activation [20,21]. These mediators activate osteoclasts, facilitating alveolar bone resorption on the pressure side. At the same time, in the tension zone, osteoblasts are activated, expressed by increased osteocalcin and alkaline phosphatase [22,23].

Nassrawin observed that OC levels in GCF vary among the 330 samples collected, which can be explained by the variation in the duration of treatment and the type of appliances used, which lead to the variation in the degree of bone remodeling [24].

The use of laser therapy during orthodontic treatment has been shown to be beneficial, through a series of clinical studies, regarding the acceleration of tooth movement, the reduction in pain and the maintenance of a healthy periodontal status [11,25,26]. The determination of markers in the crevicular fluid has been the subject of numerous studies aimed at evaluating the changes that occur in the periodontal structures during orthodontic treatment [27,28,29,30].

In our research, comparing the values obtained for OC at T1, we observed that the application of laser therapy leads to higher values of this marker, in HL compared to HC, the difference being statistically significant (*p* < 0.0001), a sign of the acceleration of bone remodeling phenomena.

Recent advances in regenerative periodontal therapy, including the use of allograft and alloplastic bone substitutes, have highlighted the importance of stimulating osteoblastic activity to enhance bone regeneration [31]. Similarly, the increase in osteocalcin levels observed after LLLT in our study supports the concept that photobiomodulation can act as a non-invasive regenerative stimulus, promoting bone remodeling and potentially accelerating post-orthodontic tissue recovery.

Bone remodeling dynamics vary with age, as bone turnover and osteoblastic activity tend to decrease over time, potentially influencing the biological response to orthodontic forces and photobiomodulation [32]. This idea could potentially be the subject of future research.

The findings of this research contribute to the understanding of how photobiomodulation influences bone metabolism and pave the way for future studies focusing on dose optimization, timing, and long-term outcomes.

### Limitations of This Study

The limitations of this study are related to the inclusion of a small number of patients, the collection of GCF samples at different stages of orthodontic treatment and the rather wide age range. Even though a wide age range (20–50) can be considered a weak point, we think that it is an advantage in order to be able to generally apply our results to further treatment guidelines. However, another limitation arises from the variability in patient age combined with the type of orthodontic treatment at the time of laser application. Both factors may influence bone metabolism and tissue response to laser biostimulation, potentially affecting the biochemical markers assessed.

## 5. Conclusions

Determining the level of OC in the gingival crevicular fluid provides an objective method for evaluating periodontal remodeling during orthodontic treatment.

In this study, higher OC levels were observed in the laser-treated hemiarches compared to the controls, suggesting that laser biostimulation may be associated with a short-term increase in bone apposition activity. However, standardized dosimetry remains essential for future clinical implementation of photobiomodulation in orthodontics. Also, since this research determined only one biochemical marker at a single time point (14 days) and did not assess tooth movement rate, pain, or post-treatment stability, further studies are needed to confirm the clinical relevance and to define standardized protocols for the use of laser biostimulation as an adjunct in orthodontic therapy.

## Figures and Tables

**Table 1:** Osteocalcin levels measured at baseline and T1 for HC and HL.

Mean Value and Standard Deviation for Identified Protein (ng/mL) T0	Mean Value and Standard Deviation for Identified Protein (ng/mL) T1-HC	Mean Value and Standard Deviation for Identified Protein (ng/mL) T1-HL
0.8507 ± 0.3237	0.8707 ± 0.1955	1.101 ± 0.2489

## Data Availability

The original contributions presented in this study are included in the article. Further inquiries can be directed to the corresponding author.
